# 4-(2-Chloro­phenyl­amino)-pent-3-en-2-one

**DOI:** 10.1107/S1600536812042043

**Published:** 2012-10-13

**Authors:** Gertruida J.S. Venter, Alice Brink, Gideon Steyl, Andreas Roodt

**Affiliations:** a Department of Chemistry, University of the Free State, PO Box 339, Bloemfontein, 9300, South Africa

## Abstract

In the title compound, C_11_H_12_ClNO, intra­molecular N—H⋯O hydrogen bonding is present. The dihedral angle between the benzene ring and the pentenone unit is 46.52 (5)°. In the crystal, C—H⋯O inter­actions between hydrogen atoms of the aryl moiety and two separate oxygen atoms occur, leading to a three-dimensional network.

## Related literature

For synthetic background and similar compounds, see: Shaheen *et al.* (2006 ▶); Venter *et al.* (2010 ▶, 2012*b* ▶). For applications, see: Brink *et al.* (2010 ▶); Pyżuk *et al.* (1993 ▶); Roodt & Steyn (2000 ▶); Tan *et al.* (2008 ▶); Xia *et al.* (2008 ▶). For related ligand systems, see: Damoense *et al.* (1994 ▶), Venter *et al.* (2012*a* ▶).
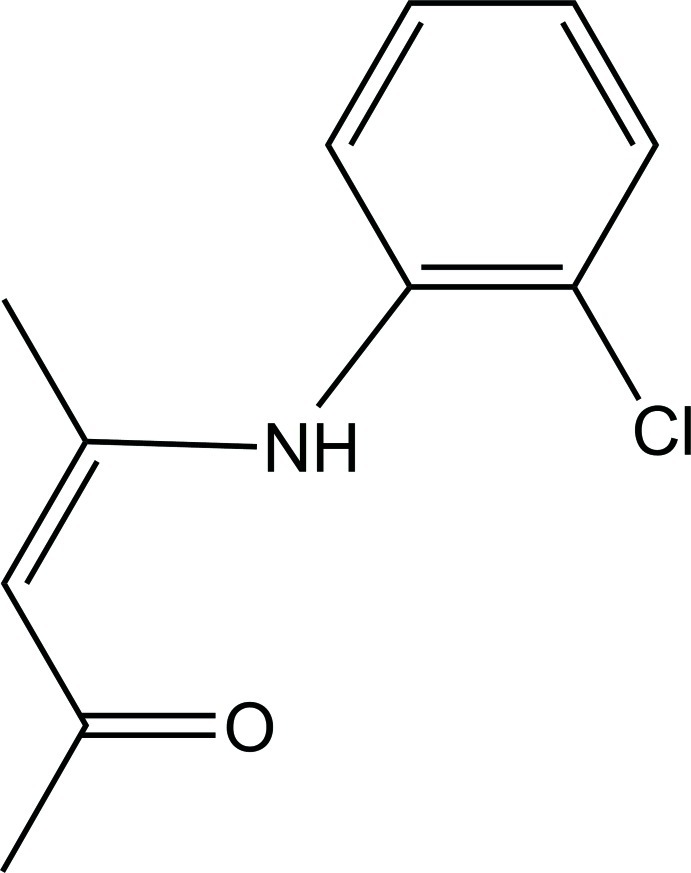


## Experimental

### 

#### Crystal data

C_11_H_12_ClNO
*M* *_r_* = 209.67
Orthorhombic, 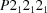

*a* = 7.3264 (3) Å
*b* = 8.7103 (4) Å
*c* = 16.1960 (7) Å
*V* = 1033.55 (8) Å^3^
*Z* = 4
Mo *K*α radiation
μ = 0.33 mm^−1^
*T* = 100 K
0.6 × 0.42 × 0.21 mm

#### Data collection

Bruker APEXII CCD area-detector diffractometer
Absorption correction: multi-scan (*SADABS*; Bruker, 2004 ▶) *T*_min_ = 0.825, *T*_max_ = 0.933
17399 measured reflections
2259 independent reflections
2211 reflections with *I* > 2σ(*I*)
*R*_int_ = 0.032

#### Refinement

*R*[*F* ^2^ > 2σ(*F* ^2^)] = 0.025
*wR*(*F* ^2^) = 0.068
*S* = 1.06
2259 reflections
126 parameters
H atoms treated by a mixture of independent and constrained refinement
Δρ_max_ = 0.21 e Å^−3^
Δρ_min_ = −0.24 e Å^−3^
Absolute structure: Flack (1983 ▶), 932 Friedel pairs
Flack parameter: 0.01 (5)

### 

Data collection: *APEX2* (Bruker, 2005 ▶); cell refinement: *SAINT-Plus* (Bruker, 2004 ▶); data reduction: *SAINT-Plus*; program(s) used to solve structure: *SHELXS97* (Sheldrick, 2008 ▶); program(s) used to refine structure: *SHELXL97* (Sheldrick, 2008 ▶); molecular graphics: *DIAMOND* (Brandenburg & Putz, 2005 ▶); software used to prepare material for publication: *WinGX* (Farrugia, 1999 ▶).

## Supplementary Material

Crystal structure: contains datablock(s) global, I. DOI: 10.1107/S1600536812042043/pk2444sup1.cif

Structure factors: contains datablock(s) I. DOI: 10.1107/S1600536812042043/pk2444Isup2.hkl

Supplementary material file. DOI: 10.1107/S1600536812042043/pk2444Isup3.cml

Additional supplementary materials:  crystallographic information; 3D view; checkCIF report

## Figures and Tables

**Table 1:** Hydrogen-bond geometry (Å, °)

*D*—H⋯*A*	*D*—H	H⋯*A*	*D*⋯*A*	*D*—H⋯*A*
N11—H11⋯O12	0.82	1.95	2.6317 (16)	139
C113—H113⋯O12^i^	0.95	2.42	3.3536 (18)	166
C115—H115⋯O12^ii^	0.95	2.43	3.3217 (18)	157

Symmetry codes: (i) 
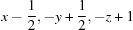
; (ii) 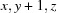.

**Fig. 1.**
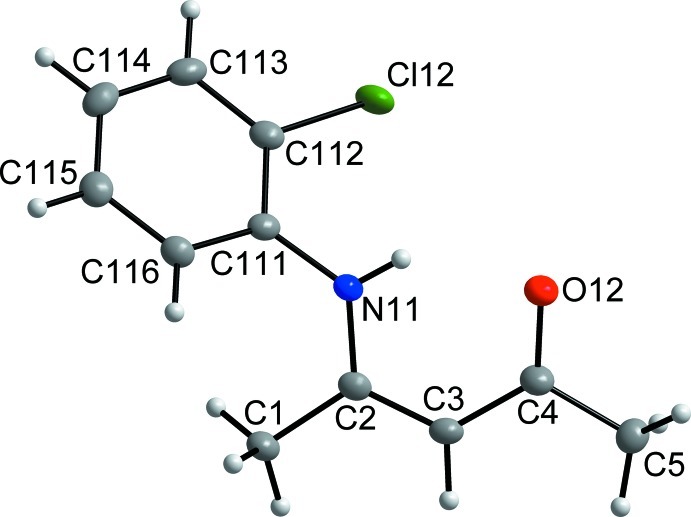
Molecular structure of the title compound. Displacement ellipsoids are drawn at the 50% probability level. Hydrogen atoms are shown as spheres of arbitrary radius.

**Fig. 2.**
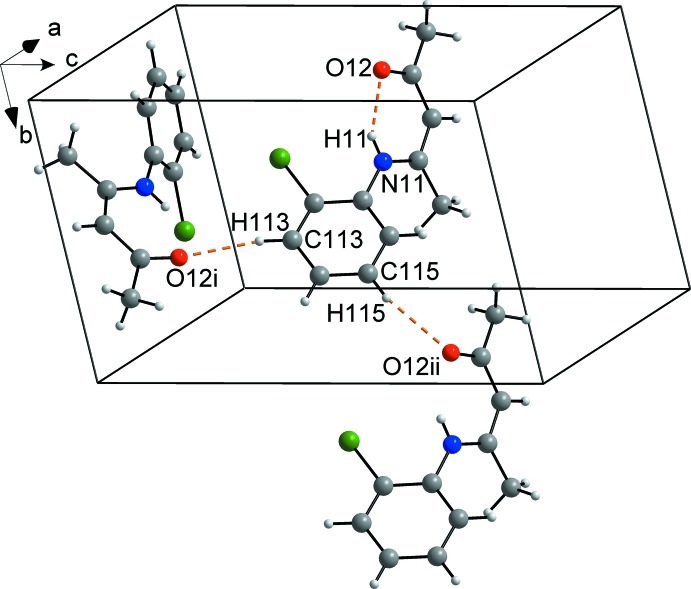
Partially filled unit cell illustrating the intra- and intermolecular hydrogen bonding interactions in the title compound, indicated with dashed lines.

## supplementary crystallographic information

### Comment

The β-diketone compound AcacH (acetylacetone; or when coordinated acetylacetonato, acac^-^) has been studied extensively, with a multitude of derivatives synthesized to date. One such derivative type, known as enaminoketones, contains both nitrogen and oxygen atoms with an unsaturated C═C bond, and is of interest in various fields including liquid crystals [Pyżuk *et al.* (1993)], fluorescence studies [Xia *et al.* (2008)], medicine [Tan *et al.* (2008)] and catalysis [Roodt & Steyn (2000); Brink *et al.* (2010)].

The title compound (Fig. 1) crystallizes in the orthorhombic space group *P*2_1_2_1_2_1_ with *Z* = 4. This enaminoketone is a derivative of 4-(phenylamino)pent-3-en-2-one [PhonyH; Shaheen *et al.* (2006)]. Bond distances differ significantly from compounds coordinated to rhodium [Venter *et al.* (2012*a*); Damoense *et al.* (1994)], but share characteristics with other enaminoketones of this type [Venter *et al.* (2010; 2012*b*). An unsaturated bond in the pentenone backbone is indicated by the difference in distance between the C_2_═C_3_ bond [1.379 (2) Å] and the C_3_–C_4_ bond [1.428 (2) Å]. The distance, N_11_···O_12_, is greatly increased (~ 0.2 Å) upon coordination. Intramolecular N_11_—H_11_···O_12_ bonding (D—A distance = 2.632 (2) Å was observed, as well as intermolecular interactions for C_113_—H_113_···O_12_i [i = x-0.5, 0.5-y, 1-z; distance = 3.3536 (18) Å] and C_115_—H_115_···O_12_ii [ii = x, y+1, z; distance = 3.3217 (18) Å]. These interactions are illustrated in Fig. 2. The dihedral angle between the benzene ring and pentenone moieties is 46.52 (5)° and is dependent on the position of the substituent on the benzene ring, where *para* substituents usually display the smallest angles (Venter *et al.*, 2010).

### Experimental

A solution of acetylacetone (11.07 g, 0.1106 mol), 2-chloro-aniline (10.73 g, 0.1008 mol) and 2 drops of H_2_SO_4_ (conc.) in 150 ml benzene was refluxed for 6 h in a Dean-Stark trap, filtered and left to crystallize. Crystals suitable for X-ray diffraction were obtained in 17.86 g (94.32%) yield. This compound is stable in air and light over a period of several months.

### Refinement

The methyl and aromatic H atoms were placed in geometrically idealized positions and constrained to ride on their parent atoms, with C—H = 0.95 Å and 0.98 Å and *U*_iso_(H) = 1.5*U*_eq_(C) and 1.2*U*_eq_(C), respectively. The methyl groups were generated to fit the difference electron density and the groups were then refined as rigid rotors.

### Crystal data

**Table:**

C_11_H_12_ClNO	*F*(000) = 440
*M**_r_* = 209.67	*D*_x_ = 1.347 Mg m^−^^3^
Orthorhombic, *P*2_1_2_1_2_1_	Mo *K*α radiation, λ = 0.71073 Å
Hall symbol: P 2ac 2ab	Cell parameters from 5717 reflections
*a* = 7.3264 (3) Å	θ = 2.7–28.3°
*b* = 8.7103 (4) Å	µ = 0.33 mm^−^^1^
*c* = 16.1960 (7) Å	*T* = 100 K
*V* = 1033.55 (8) Å^3^	Cuboid, yellow
*Z* = 4	0.6 × 0.42 × 0.21 mm

### Data collection

**Table:**

Bruker APEXII CCD area-detector diffractometer	2259 independent reflections
Radiation source: fine-focus sealed tube	2211 reflections with *I* > 2σ(*I*)
Graphite monochromator	*R*_int_ = 0.032
φ and ω scans	θ_max_ = 27.0°, θ_min_ = 2.5°
Absorption correction: multi-scan (*SADABS*; Bruker, 2004)	*h* = −9→9
*T*_min_ = 0.825, *T*_max_ = 0.933	*k* = −11→11
17399 measured reflections	*l* = −20→19

### Refinement

**Table:**

Refinement on *F*^2^	Secondary atom site location: difference Fourier map
Least-squares matrix: full	Hydrogen site location: inferred from neighbouring sites
*R*[*F*^2^ > 2σ(*F*^2^)] = 0.025	H atoms treated by a mixture of independent and constrained refinement
*wR*(*F*^2^) = 0.068	*w* = 1/[σ^2^(*F*_o_^2^) + (0.0376*P*)^2^ + 0.2837*P*] where *P* = (*F*_o_^2^ + 2*F*_c_^2^)/3
*S* = 1.06	(Δ/σ)_max_ = 0.013
2259 reflections	Δρ_max_ = 0.21 e Å^−^^3^
126 parameters	Δρ_min_ = −0.24 e Å^−^^3^
0 restraints	Absolute structure: Flack (1983), 932 Friedel pairs
Primary atom site location: structure-invariant direct methods	Flack parameter: 0.01 (5)

### Special details

**Table:**

Geometry. All s.u.'s (except the s.u. in the dihedral angle between two l.s. planes) are estimated using the full covariance matrix. The cell s.u.'s are taken into account individually in the estimation of s.u.'s in distances, angles and torsion angles; correlations between s.u.'s in cell parameters are only used when they are defined by crystal symmetry. An approximate (isotropic) treatment of cell s.u.'s is used for estimating s.u.'s involving l.s. planes.
Refinement. Refinement of *F*^2^ against ALL reflections. The weighted *R*-factor *wR* and goodness of fit *S* are based on *F*^2^, conventional *R*-factors *R* are based on *F*, with *F* set to zero for negative *F*^2^. The threshold expression of *F*^2^ > 2σ(*F*^2^) is used only for calculating *R*-factors(gt) *etc*. and is not relevant to the choice of reflections for refinement. *R*-factors based on *F*^2^ are statistically about twice as large as those based on *F*, and *R*- factors based on ALL data will be even larger.

### Fractional atomic coordinates and isotropic or equivalent isotropic displacement parameters (Å^2^)

**Table:**

	*x*	*y*	*z*	*U*_iso_*/*U*_eq_	
Cl12	0.00235 (5)	0.20519 (4)	0.531562 (19)	0.02339 (10)	
N11	0.21805 (15)	0.28866 (14)	0.67897 (7)	0.0174 (2)	
H11	0.2500 (8)	0.209 (2)	0.6556 (6)	0.027 (5)*	
O12	0.33003 (14)	0.00171 (12)	0.68371 (6)	0.02136 (18)	
C3	0.24678 (18)	0.14426 (16)	0.80149 (9)	0.0178 (3)	
H3	0.2362	0.1411	0.8599	0.021*	
C2	0.20915 (17)	0.28095 (16)	0.76209 (8)	0.0169 (3)	
C111	0.18055 (18)	0.41335 (16)	0.62662 (8)	0.0166 (3)	
C116	0.2464 (2)	0.56091 (16)	0.64159 (9)	0.0197 (3)	
H116	0.3191	0.5797	0.6891	0.024*	
C4	0.3006 (2)	0.00740 (18)	0.75983 (9)	0.02136 (18)	
C113	0.04201 (19)	0.50725 (19)	0.49974 (9)	0.0229 (3)	
H113	−0.0264	0.4881	0.451	0.027*	
C5	0.3168 (2)	−0.13853 (17)	0.80936 (9)	0.0227 (3)	
H5A	0.2032	−0.1974	0.8047	0.034*	
H5B	0.339	−0.1131	0.8674	0.034*	
H5C	0.4185	−0.2	0.7881	0.034*	
C112	0.08035 (18)	0.38927 (17)	0.55421 (9)	0.0184 (3)	
C115	0.20717 (19)	0.68074 (17)	0.58791 (9)	0.0226 (3)	
H115	0.2505	0.7812	0.5996	0.027*	
C1	0.1554 (2)	0.41966 (16)	0.81135 (9)	0.0203 (3)	
H1A	0.2628	0.4845	0.8205	0.03*	
H1B	0.1056	0.3871	0.8647	0.03*	
H1C	0.0627	0.478	0.7811	0.03*	
C114	0.1048 (2)	0.65395 (18)	0.51727 (9)	0.0243 (3)	
H114	0.0776	0.7363	0.4808	0.029*	

### Atomic displacement parameters (Å^2^)

**Table:**

	*U*^11^	*U*^22^	*U*^33^	*U*^12^	*U*^13^	*U*^23^
Cl12	0.02513 (17)	0.02720 (17)	0.01784 (17)	−0.00589 (15)	−0.00152 (14)	−0.00549 (12)
N11	0.0194 (5)	0.0181 (5)	0.0146 (5)	0.0016 (5)	−0.0004 (4)	−0.0024 (5)
O12	0.0232 (4)	0.0229 (4)	0.0180 (4)	−0.0007 (3)	0.0032 (3)	−0.0004 (3)
C3	0.0158 (6)	0.0234 (7)	0.0140 (7)	−0.0011 (5)	−0.0006 (5)	−0.0012 (5)
C2	0.0122 (6)	0.0218 (6)	0.0167 (6)	−0.0010 (5)	−0.0007 (5)	−0.0026 (5)
C111	0.0136 (6)	0.0216 (6)	0.0145 (6)	0.0013 (5)	0.0015 (5)	−0.0012 (5)
C116	0.0168 (6)	0.0224 (6)	0.0198 (7)	−0.0008 (5)	0.0010 (5)	−0.0028 (5)
C4	0.0232 (4)	0.0229 (4)	0.0180 (4)	−0.0007 (3)	0.0032 (3)	−0.0004 (3)
C113	0.0202 (7)	0.0333 (7)	0.0152 (6)	0.0025 (6)	0.0003 (5)	0.0019 (6)
C5	0.0211 (6)	0.0230 (7)	0.0241 (8)	0.0020 (6)	0.0011 (6)	0.0025 (6)
C112	0.0151 (6)	0.0236 (7)	0.0165 (7)	−0.0019 (5)	0.0026 (5)	−0.0027 (5)
C115	0.0212 (7)	0.0224 (7)	0.0242 (7)	−0.0013 (6)	0.0062 (5)	0.0008 (6)
C1	0.0212 (6)	0.0221 (7)	0.0175 (7)	0.0018 (5)	0.0007 (5)	−0.0032 (5)
C114	0.0228 (7)	0.0276 (7)	0.0224 (8)	0.0032 (6)	0.0042 (6)	0.0077 (6)

### Geometric parameters (Å, º)

**Table:**

Cl12—C112	1.7412 (15)	C4—C5	1.508 (2)
N11—C2	1.3494 (18)	C113—C112	1.383 (2)
N11—C111	1.4049 (18)	C113—C114	1.387 (2)
N11—H11	0.8243	C113—H113	0.95
O12—C4	1.2524 (18)	C5—H5A	0.98
C3—C2	1.3787 (19)	C5—H5B	0.98
C3—C4	1.425 (2)	C5—H5C	0.98
C3—H3	0.95	C115—C114	1.388 (2)
C2—C1	1.5005 (19)	C115—H115	0.95
C111—C116	1.3942 (19)	C1—H1A	0.98
C111—C112	1.3994 (19)	C1—H1B	0.98
C116—C115	1.389 (2)	C1—H1C	0.98
C116—H116	0.95	C114—H114	0.95
			
C2—N11—C111	129.12 (13)	C4—C5—H5A	109.5
C2—N11—H11	115.4	C4—C5—H5B	109.5
C111—N11—H11	115.4	H5A—C5—H5B	109.5
C2—C3—C4	123.95 (13)	C4—C5—H5C	109.5
C2—C3—H3	118	H5A—C5—H5C	109.5
C4—C3—H3	118	H5B—C5—H5C	109.5
N11—C2—C3	119.67 (12)	C113—C112—C111	121.98 (13)
N11—C2—C1	120.21 (13)	C113—C112—Cl12	118.89 (11)
C3—C2—C1	120.11 (12)	C111—C112—Cl12	119.13 (11)
C116—C111—C112	117.74 (13)	C114—C115—C116	120.12 (13)
C116—C111—N11	122.69 (12)	C114—C115—H115	119.9
C112—C111—N11	119.50 (12)	C116—C115—H115	119.9
C115—C116—C111	120.82 (13)	C2—C1—H1A	109.5
C115—C116—H116	119.6	C2—C1—H1B	109.5
C111—C116—H116	119.6	H1A—C1—H1B	109.5
O12—C4—C3	123.13 (14)	C2—C1—H1C	109.5
O12—C4—C5	118.48 (14)	H1A—C1—H1C	109.5
C3—C4—C5	118.35 (13)	H1B—C1—H1C	109.5
C112—C113—C114	119.11 (14)	C113—C114—C115	120.18 (14)
C112—C113—H113	120.4	C113—C114—H114	119.9
C114—C113—H113	120.4	C115—C114—H114	119.9
			
C111—N11—C2—C3	177.99 (12)	C114—C113—C112—C111	0.1 (2)
C111—N11—C2—C1	−0.9 (2)	C114—C113—C112—Cl12	−179.63 (11)
C4—C3—C2—N11	1.8 (2)	C116—C111—C112—C113	−1.8 (2)
C4—C3—C2—C1	−179.31 (13)	N11—C111—C112—C113	−178.91 (12)
C2—N11—C111—C116	46.2 (2)	C116—C111—C112—Cl12	177.84 (10)
C2—N11—C111—C112	−136.84 (15)	N11—C111—C112—Cl12	0.77 (17)
C112—C111—C116—C115	2.6 (2)	C111—C116—C115—C114	−1.5 (2)
N11—C111—C116—C115	179.53 (13)	C112—C113—C114—C115	1.1 (2)
C2—C3—C4—O12	4.7 (2)	C116—C115—C114—C113	−0.3 (2)
C2—C3—C4—C5	−173.14 (12)		

### Hydrogen-bond geometry (Å, º)

**Table:**

*D*—H···*A*	*D*—H	H···*A*	*D*···*A*	*D*—H···*A*
N11—H11···O12	0.82	1.95	2.6317 (16)	139
C113—H113···O12^i^	0.95	2.42	3.3536 (18)	166
C115—H115···O12^ii^	0.95	2.43	3.3217 (18)	157

Symmetry codes: (i) *x*−1/2, −*y*+1/2, −*z*+1; (ii) *x*, *y*+1, *z*.
